# Fruit Season

**Published:** 1858-06

**Authors:** 


### FRUIT SEASON.

LOVELY June has come, and the delicious Strawberry will soon be here, to be followed in succession by other berries and fruits, until the Fall, showing at once the wisdom and beneficence of our common Father. How to use them wisely, and thus derive the fullest advantage from that wise beneficence, it is worth while to know. The earlier in the day fruits are eaten the better: they should be ripe, fresh, and perfect, and eaten in their natural state, with the important advantage of its being almost impossible to take too many; their healthful qualities depend on their ripe acidity, but if sweetened with sugar, the acidity is not only neutralized, but the stomach is tempted to receive more than it is possible to digest, and if cream is taken with them, the labor of digestion is increased: hence the fearful attacks of cholera morbus which sometimes follow the free use of fruits and berries with sugar and cream.

No liquid of any description should be drank within an hour after eating fruits, nor should anything else be eaten within two or three hours after—thus time being allowed for them to pass out of the stomach, the system derives from them all their enlivening, cooling, and opening influences. The great rule is, eat fruits and berries while fresh, ripe, and perfect, in their natural state, without eating or drinking anything for at least two hours afterwards. With these restrictions, fruits and berries may be eaten in moderation during any hour of the day, and without getting tired of them, or ceasing to be benefited by them during the whole season. It is a great waste of lusciousness that fruits and berries, in their natural state, are not made the sole dessert at our meals, for three-fourths of the year; human enjoyment and health, and even life, would be promoted by it.

---

THE AILANTHUS, luxuriant and wholly free from worms, is declared by Dr. HILDRETH to be innocent of the unhealthful odors attributed to it—they arise, he says, from the worms on neighboring trees; others say they come only from the staminate, or male flower.

